# Slandering Doctors

**Published:** 1882-08

**Authors:** 


### SLANDERING DOCTORS.

A GREAT many jokes are cracked at the expense of the doctors, *and* at the expense of the reputation for intellect of those who crack them; for a moment's consideration, which, by the way, in this fast age, is not given to anything of true importance, except by the few—a moment's consideration would teach any one, that it is to the doctor's interest to keep the patient alive as long as possible, for *as long as the patient lives*, *he pays!* Witness the desperate efforts made to protract life for a few hours, in the last extremity; how the medicine is poured down every five minutes, as long as the dying man can swallow; how the blister plaster encases ankle, wrist, and waist, to kindle up again the powers of life, for, with returning life, returns the prospect of dollars. For our part, we could never appreciate the philosophy of torturing the poor dying body in the ways just alluded to, to the last moment of existence. The great Washington prayed to be allowed to die in peace. When our last hour comes, hoist the window, throw the door wide open, without a draft; moisten the lips; clear the room of all but one or two; let all the pure air possible, get to the laboring lungs.

